# Predicting nonlinear physical aging of glasses from equilibrium relaxation via the material time

**DOI:** 10.1126/sciadv.abl9809

**Published:** 2022-03-16

**Authors:** Birte Riechers, Lisa A. Roed, Saeed Mehri, Trond S. Ingebrigtsen, Tina Hecksher, Jeppe C. Dyre, Kristine Niss

**Affiliations:** “Glass and Time”, IMFUFA, Department of Science and Environment, Roskilde University, P.O. Box 260, DK-4000 Roskilde, Denmark.

## Abstract

The noncrystalline glassy state of matter plays a role in virtually all fields of materials science and offers complementary properties to those of the crystalline counterpart. The caveat of the glassy state is that it is out of equilibrium and therefore exhibits physical aging, i.e., material properties change over time. For half a century, the physical aging of glasses has been known to be described well by the material-time concept, although the existence of a material time has never been directly validated. We do this here by successfully predicting the aging of the molecular glass 4-vinyl-1,3-dioxolan-2-one from its linear relaxation behavior. This establishes the defining property of the material time. Via the fluctuation-dissipation theorem, our results imply that physical aging can be predicted from thermal-equilibrium fluctuation data, which is confirmed by computer simulations of a binary liquid mixture.

## Teaser

Equilibrium relaxation behavior predicts the nonlinear physical aging of glasses.

## INTRODUCTION

Physical aging deals with small property changes resulting from molecular rearrangements (*1*–*5*). While the aging of a material is, in practice, often due to chemical degradation, physical aging does not involve any chemical change. Understanding this type of aging is crucial for applications of noncrystalline materials such as oxide glasses (*4*, *6*–*8*), polymers (*2*, *5*, *9*–*13*), metallic glasses (*14*–*18*), amorphous pharmaceuticals (*19*), colloidal suspensions (*20*), etc. For instance, the performance of a smartphone display glass substrate is controlled by details of the physical aging during production (*21*), and some plastics eventually become brittle as a result of physical aging (*22*). Noncrystalline or partly noncrystalline states play a role in modern materials science, e.g., in connection with metal-organic frameworks (*23*) and high-entropy alloys (*24*), and physical aging is also important in connection with active matter (*25*–*27*). Last, it should be mentioned that aging under nanoconfined conditions differs from that of bulk materials (*28*). The lack of a fundamental understanding of the glassy state and its aging with time influences all branches of materials science, which explains the continued interest in the field from a theoretical point of view (*17*, *27*, *29*–*32*).

Describing and predicting physical aging has been a focus of glass science for many years, yet the subject still presents important challenges (*13*, *31*). In this work, we address the concept of a material (“reduced”) time controlling aging, which was proposed by Narayanaswamy in 1971 in a paper dealing with the physical aging of oxide glasses (*6*). A closely related formalism describing polymer aging was developed a few years later by Kovacs and co-workers (*3*) and, in the 1990s, in the entirely different context of spin glasses by Cugliandolo and Kurchan (*29*). The material-time concept rationalizes several notable aging phenomena (*4*, *6*, *33*–*35*). It is used routinely in both basic research and applications. The material-time formalism is generally recognized to describe well the physical aging of systems subjected to relatively small temperature variations, but the existence of a material time has never been validated in direct experiments. We do this here in long-time experiments on a glass-forming molecular liquid by demonstrating the fundamental prediction that linear response aging data determine the nonlinear aging behavior in the intermediate regime involving temperature variations of a few percent.

Physical aging is a complex phenomenon as it is both nonexponential and nonlinear. The simplest and best controlled aging experiment is based on the temperature jump protocol: The sample is initially in a state of thermal equilibrium, then its temperature is changed instantaneously, i.e., rapidly compared to the response time of the material, and the full approach to equilibrium at the new temperature is monitored as a function of time (*36*). This procedure requires a setup that allows for fast temperature changes and has a precise temperature control with a minimal long-time drift. Moreover, accurate measurements are needed because the long time tail of physical aging, as well as the entire aging response to a small temperature step, involves only minute changes of material properties.

Our experimental setup is based on a Peltier element in direct contact with a plane-plate capacitor. The setup keeps temperature constant over months with less than 1 mK variation, and the samples are so thin (50 μm) that the temperature may be changed within a few seconds to a new, constant value. Dielectric properties are monitored using an ultraprecision Andeen-Hagerling capacitance bridge. More details on the setup are provided in Materials and Methods and in (*36*–*40*).

## RESULTS

**Fig. 1.**
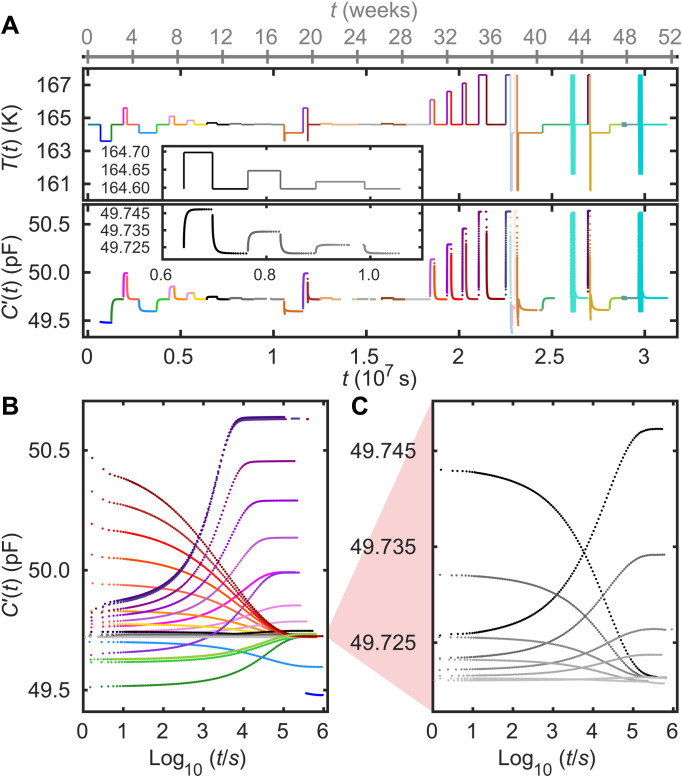
Overview of the temperature protocol and the raw data of the full experiment on VEC. (**A**) The experimental protocol realized by temperature modulations around the reference temperature 164.6 K (top) and the real part of the measured capacitance *C*′(10 kHz) (bottom), both plotted as functions of time on a linear scale. Jumps larger than 100 mK are colored, while jumps of 100 mK or less are depicted on a gray scale; a selection of the latter is shown in the inset. The sinusoidal temperature modulations that are also studied (see below) are not resolved in this figure, where they appear as turquoise thick vertical lines. (**B**) The capacitance *C*^′^(10 kHz) data plotted as functions of the logarithm of the time *t* that has passed after each jump. (**C**) Magnification of the jumps of magnitude 100 mK or less.

We performed several temperature jump experiments around a reference temperature on the glass-forming liquid 4-vinyl-1,3-dioxolan-2-one (VEC) and monitored, after each jump, both the real and the imaginary part of the capacitance at 10 kHz as the system gradually equilibrates (*41*, *42*). The real part of the VEC data is presented in Fig. 1; the imaginary part of the data can be found in the Supplementary Materials in which we also give analogous data for *N*-methyl-ε-caprolactam (NMEC). Capacitance can be measured very precisely and is an excellent probe in aging experiments (*36*, *43*, *44*). For samples of molecules with a low dipole moment, the real part of the capacitance provides a direct measure of the density (*38*). The VEC and NMEC molecules have large dipole moments, which implies that rotational polarizations contribute substantially to the capacitance even at high frequencies (*38*, *45*). Although this means that the simple connection to density is lost, the capacitance still provides a precise probe of the state of the sample during aging.

**Fig. 2.**
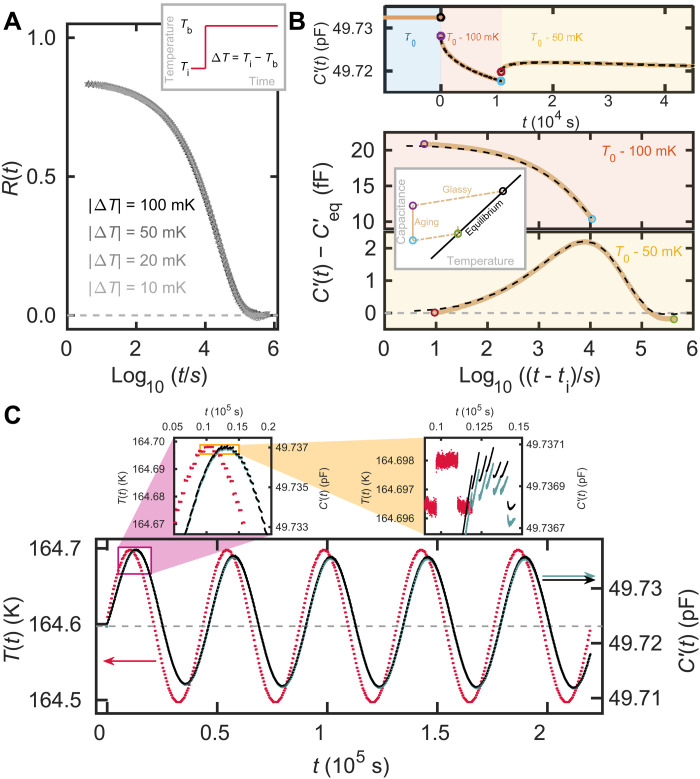
The real part of the capacitance from small-amplitude temperature modulation experiments on VEC along with predictions demonstrating that the response is linear. (**A**) Normalized relaxation function (Eq. 3) of single temperature jumps of amplitude 10 to 100 mK around the reference temperature *T*_0_ = 164.6 K. All data collapse as predicted for linear relaxation. (**B**) Data from a small-amplitude temperature double jump starting at *T*_0_ = 164.6 K and jumping first by −100 mK and then by +50 mK (colored curves) along with the prediction according to Eq. 4 (black dashed lines). The top panel shows the full experiment on a linear time scale; the bottom panel shows the data on a logarithmic time scale that sets the time of the beginning of each temperature jump to zero. The inset illustrates the temperature protocol. (**C**) Sinusoidal small-amplitude temperature protocol (red points) and data (turquoise points behind the black points). The prediction based on the linear response formalism (Eq. 5) is shown as black points. The deviations between prediction and data that can barely be discerned in the main figure can be seen in the magnified panels.

**Fig. 3.**
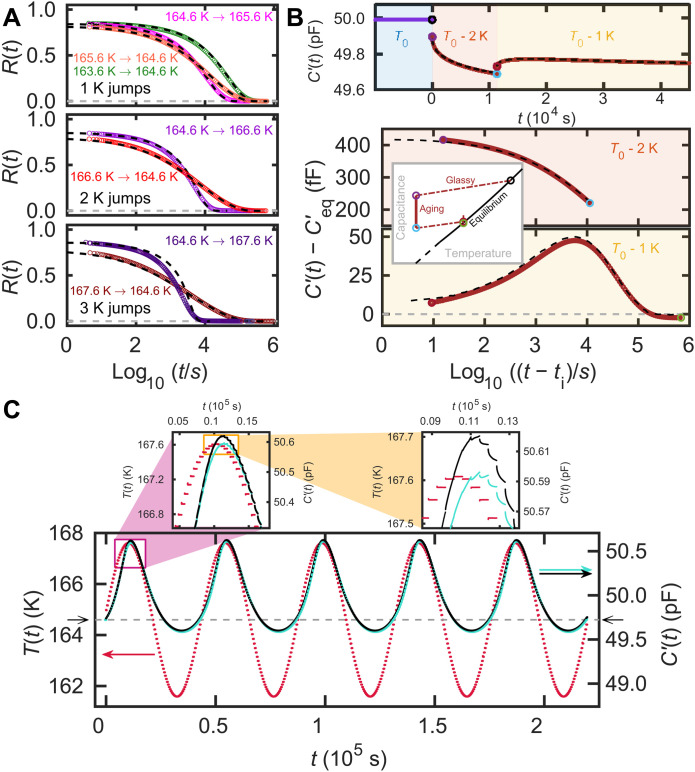
The real part of the capacitance from large-amplitude temperature modulation experiments on VEC along with predictions based on the measured linear response *R*_lin_(*t*). The prediction is calculated using Eq. 6 in combination with Eqs. 7 and 8. Colored curves are the data, and black dashed lines are predictions. (**A**) Normalized relaxation functions (Eq. 3) of single temperature jumps with amplitudes ranging from 1 to 3 K. (**B**) Data from a double jump starting at *T*_0_ = 167.6 K and jumping by −2 K and +1 K (colored curves). The top panel shows the full experiment on a linear time scale; the bottom panel shows the data on a logarithmic time scale, setting the time of the beginning of each temperature jump to zero. The inset illustrates the temperature protocol. (**C**) Sinusoidal temperature protocol (red points) and data (turquoise points) shown together. The horizontal dashed line marks the equilibrium capacitance at the starting temperature 164.6 K. The prediction of the material-time formalism is shown as black points. The response is highly nonlinear, resulting in a nonsinusoidal curve that is far from symmetric around the horizontal dashed line. The deviations between prediction and data that can barely be discerned in the main panel are visible in the magnified panels.

The reference temperature for the VEC experiment is 164.6 K at which the main (alpha) relaxation time is roughly 12 hours (see the Supplementary Materials). This is large enough for the setup to thermalize after a temperature jump before any notable relaxation has taken place in the sample. Figure 1A shows our temperature protocol with the 10 kHz real part of the capacitance measured as a function of time. The first 36 weeks of the experiment were devoted to single temperature jumps with size varying from 10 mK to 3 K, carefully equilibrating the sample after each jump before the next one was initiated. The last 15 weeks were spent on temperature variations involving double jumps and sinusoidal modulations. The latter are not resolved in this figure, where they appear as thick turquoise vertical lines; we return to these protocols later (Figs. 2 and 3). Figure 1B shows the data for the single jumps plotted as a function of the logarithm of the time that has passed after each jump was initiated. Note that these curves have very different shapes, demonstrating that even fairly small temperature jumps lead to a notable nonlinear response. This is a hallmark of physical aging, reflecting the “asymmetry of approach” that jumping to the same final temperature from a higher temperature results in a faster and more stretched response than the same size jump coming from below (*4*, *6*, *13*, *46*). Figure 1C focuses on the smaller jumps that are not resolved in Fig. 1B.

The response to a temperature variation is usually highly nonlinear. Nevertheless, any response is expected to have a small-amplitude limit at which the measured quantity, *X*(*t*), depends linearly on the external perturbation. Even in this limit, the measured signal, in general, depends on the temperature history. This means that *X*(*t*) in the linear limit is given by a convolution of the change in temperature with the normalized linear time-domain response function *R*_lin_(*t*) in the following manner

$$X(t)-X_{\text{eq}}(T)=-\alpha_{X}\int_{-\infty }^{t}R_{\text{lin}}(t-t\prime )\frac{\mathrm{dT}}{dt\prime }dt\prime$$ (1)

Here, the temperature *T* is, in general, a function of the time *t*, *X*_eq_(*T*) is the equilibrium value of the measured property at temperature *T*, and α*_X_* = *dX*_eq_/*dT* quantifies its temperature dependence. This linear description is also known as the Boltzmann superposition principle. In the case of a temperature jump at time zero from the initial temperature *T*_i_ to the “bath” temperature *T*_b_, one has *dT*/*dt*′ = −Δ*T*δ(*t*′) in which (following the convention in the field) Δ*T* = *T*_i_ − *T*_b_ and δ(*t*′) is the Dirac delta function. Equation 1 implies that the time-dependent response is α*_X_*Δ*TR*_lin_(*t*). Defining Δ*X* = *X*_eq_(*T*_i_) − *X*_eq_(*T*_b_) and noting that Δ*X* = α*_X_*Δ*T*, the response is given by

$$X(t)-X_{\text{eq}}(T_{b})=\Delta XR_{\text{lin}}(t)$$ (2)

We have previously worked with this linear limit for temperature jumps down to 100 mK (*38*–*40*, *47*); the data of the present paper take this a step further by involving temperature jumps as small as 10 mK, as well as by optimizing the protocol to make it possible to properly resolve both the long- and the short-time plateaus of the linear aging curve.

Linearity is investigated, in general, by considering the normalized relaxation function of the quantity *X*, denoted by *R*(*t*), which for a jump to temperature *T*_b_ at *t* = 0 is defined by

$$R(t)=\frac{X(t)-X_{\text{eq}}(T_{b})}{\Delta X}$$ (3)

We note that *R*(0) = 1 and that *R*(*t*) goes to zero as the system equilibrates at *T*_b_ at long times. Whenever the data are in the linear regime, the relaxation function is the response function of Eq. 1, *R*(*t*) = *R*_lin_(*t*), i.e., relaxations following all temperature jumps have the same time-dependent normalized relaxation function in the linear limit.

Figure 2 shows *R*(*t*) for temperature jumps of magnitude 10 to 100 mK to and from the reference temperature, where *X* = *C*′(10 kHz) is the real part of the capacitance at 10 kHz of VEC. Similar data are shown for the imaginary part and for NMEC in the Supplementary Materials. The short-time plateau of *R*(*t*) is below the theoretical value *R*(0) = 1. This is because there is a fast response that cannot be resolved by our setup, a common finding in studies of physical aging that was discussed in detail in previous works (*39*, *47*). We believe that the fast response mainly happens on the phonon time scale due to vibrational and librational equilibration. In addition, one or more beta relaxations may take place at times shorter than the experimental cutoff of about 4 s (the time it takes to change temperature and equilibrate the setup at the new temperature). However, in the dielectric spectra (Fig. S1), the beta relaxation is only seen as a small shoulder, which indicates that it probably only accounts for a few percent of the initial decay in *R*(*t*).

All the normalized relaxation functions observed for these small temperature jumps around the reference temperature collapse within the experimental uncertainty, as predicted for a linear response. True linearity is a theoretical limit, which means that higher precision and better resolution would reveal tiny differences between the relaxation curves. For the data of Fig. 2, the uncertainty is of the same order of magnitude as the symbol size and no differences are resolved, meaning that the measured curves for all practical purposes represent the linear response function *R*_lin_(*t*). In the following, we demonstrate how the linear response function can be used to predict the response for different temperature protocols resulting in linear (Fig. 2) and nonlinear (Fig. 3) aging responses.

A simple generalization of the temperature jump experiment is to introduce a second jump before the system has equilibrated fully in response to the first one, a so-called double-jump experiment. If the temperature changes are both small enough to be within the linear range, then the measured value of *X*(*t*) after the second jump is a sum of the responses to the individual jumps. For two temperature jumps corresponding to changes in the measured property *X* by Δ*X*_1_ and Δ*X*_2_ performed at times *t*_1_ and *t*_2_ (*t*_1_ < *t*_2_), respectively, one has

$$X(t)=\Delta X_{1}R_{\text{lin}}(t-t_{1})+\Delta X_{2}R_{\text{lin}}(t-t_{2})+X_{\text{eq}}(T_{2})\;\text{for}\;t>t_{2}$$ (4)

where *T*_2_ is final temperature after the second jump.

We test Eq. 4 for the Ritland-Kovacs crossover protocol (*4*, *46*, *48*, *49*) consisting of two consecutive temperature jumps with an opposite sign determined such that the observable *X* has its equilibrium value right after the second jump. Figure 2B illustrates this protocol and shows the observations after a −100 mK jump followed by a +50 mK jump for VEC. The data reproduce the crossover effect that *X*(*t*) exhibits a peak after the second jump (*4*, *46*). This bump is a manifestation of the memory present for any nonexponential linear response (*4*). Along with the data, the predictions based on Eq. 4 and the measured *R*_lin_(*t*) from the 50 mK jump in Fig. 2A are also shown. The prediction collapses almost exactly with the double-jump data. These small-amplitude double jump results provide an extra confirmation that we have reached the linear limit of physical aging. Similar data are presented for NMEC in the Supplementary Materials, which also provides data for more VEC small (linear) jumps.

Moving on from the double temperature jump, we note that Eq. 1 predicts the response to any temperature perturbation small enough to be linear. Because we do not have an analytic expression for *R*_lin_(*t*), the integral is calculated by generalizing the sum in Eq. 4

$$X(t)=\sum_{i=1}^{N}\Delta X_{i}R_{\text{lin}}(t-t_{i})+X_{\text{eq}}(T_{N})\;\text{for}\;t>t_{N}$$ (5)

where *T_N_* is the final temperature after *N* jumps. In Fig. 2C, we show how Eq. 5 predicts the output of a small sinusoidal temperature perturbation. The frequency of the perturbation is 2.3 × 10^−5^ Hz, which is the inverse of the estimated equilibrium relaxation time of the sample at the reference temperature 164.6 K. The amplitude is 100 mK, i.e., within the linear regime of single jumps. The prediction follows the data with a high accuracy, including both the transient behavior (seen, e.g., in a first peak that is higher than the second) and the phase shift. Tiny deviations between prediction and data can be seen in the inset, which also shows how the temperature protocol is composed of 2-mK temperature steps.

After establishing the linear aging limit and showing how linear temperature jump data can be used to predict the response of other linear temperature protocols, we now turn to the main result of this paper, a proof of the existence of a material time for VEC. The radically new idea in the 1970s (*3*, *6*) was that aging becomes linear when it is described in terms of the material time ξ(*t*) instead of the laboratory time *t*. One assumes the so-called time aging-time superposition, meaning that the spectral shape of *R*_lin_ is independent of the state of the sample. As a consequence of these assumptions, Eqs. 1, 4, and 5 describe also nonlinear experiments by replacing the laboratory time with the material time, i.e.,

$$X(\xi )=\sum_{i=1}^{N}\Delta X_{i}R_{\text{lin}}(\xi -\xi_{i})+X_{\text{eq}}(T_{N})\;\text{for}\;\xi >\xi_{N}$$ (6)

The material time is “measured” by a clock with a rate that reflects the state of the sample, and the nonlinearity of physical aging is a consequence of this fact (*3*, *6*, *29*, *50*). The material time may be thought of as analogous to the proper time in the theory of relativity, which is the time recorded on a clock following the observer. Although a microscopic definition of the material time remains elusive, this concept is generally recognized to form the basis of a good description of physical aging involving relatively small temperature variations (*4*). The very fundamental assumption of the formalism, however, that nonlinear aging phenomena can be predicted from the linear aging limit has never been validated. In the following, we do so by showing how the measured linear response determines the response to nonlinear temperature protocols for VEC and, in the Supplementary Materials, for NMEC.

Using Eq. 6 requires a connection between the laboratory time *t* and the material time ξ. This is obtained by introducing the time-dependent aging rate γ(*t*) defined (*3*, *6*, *8*, *11*, *51*) by

γ(t)=dξ(t)/dt (7)

In equilibrium, the aging rate equals the relaxation rate γ_eq_ defined as the inverse of the equilibrium relaxation time. Thus, a linear experiment is the limiting case for which the aging rate is constant and the material time is proportional to the laboratory time, ξ_lin_(*t*) = γ_eq_*t*.

Different strategies have been used to estimate γ(*t*) during aging, often via the so-called fictive temperature (*4*, *33*, *35*, *48*). We here adopt the single-parameter aging ansatz (*4*, *6*, *52*, *53*, *69*) according to which the aging rate is controlled by the measured quantity *X*(*t*) itself. In the simplest realization, single-parameter aging is characterized by (*53*)

$$\text{log}\;(\gamma (t))-\text{log}\;(\gamma_{\text{eq}}(T))=\Lambda (X(t)-X_{\text{eq}}(T))$$ (8)

Here, γ_eq_(*T*) and *X*_eq_(*T*) are the equilibrium values of γ and *X* at the temperature *T*, and Λ is a constant that depends only on the substance and the monitored property *X*. It should be noted that Eq. 8 is arrived at by first-order Taylor expansions and, for this reason, can only be expected to apply for relatively small temperature variations.

The material-time description in Eq. 6, combined with Eqs. 7 and 8, gives a unique prediction for *X*(*t*) for any temperature protocol. Equation 6 predicts the value of *X*(ξ), while Eqs. 7 and 8 connect the material and laboratory times by stretching or compressing the time scale axis. The input needed for the prediction is *R*_lin_(*t*) as determined in Fig. 2A, the equilibrium values of the rate γ_eq_(*T*) and of the measured property *X*_eq_(*T*), and the parameter Λ. We have equilibrium measurements of *X*_eq_(*T*) down to 163.6 K and have extrapolated values to lower temperatures (see the Supplementary Materials). The values used for γ_eq_(*T*) are extrapolations from a fit of relaxation times derived from dielectric spectra, which, down to 163.6 K, are proportional to the aging rates (see the Supplementary Materials).

The parameter Λ is determined by the method described in (*53*) from the two temperature jump experiments of magnitude ±1 K to the reference temperature 164.6 K (see the Supplementary Materials). This Λ value was used for predicting all other nonlinear responses. Figure 3A shows *R*(*t*) data from the nonlinear single-temperature jumps. It is seen that the short-time plateaus of *R*(*t*) for the different jumps do not coincide. This is due to a difference in the short-time relaxation deriving from the response on the phonon time scale and, possibly, also from one or more beta relaxations. To predict the aging, we have adjusted for this difference in a manner where the short-time decay of *R*(*t*) depends on both the initial and final temperatures (see the Supplementary Materials).

Figure 3 reports the main results of the paper: data from nonlinear temperature protocols along with predictions based on the linear temperature jump data. The nonlinear protocols mirror the linear protocols of Fig. 2. Figure 3A shows single temperature jumps, Fig. 3B shows a −2 K and +1 K double jump, and Fig. 3C shows a sinusoidal temperature modulation with amplitude 3 K and the same frequency as the linear sinusoidal protocol of Fig. 2.

The single jumps in Fig. 3A exhibit the asymmetry of approach characteristic of nonlinear aging (*13*, *46*): “self-acceleration” of up jumps where the relaxation rate speeds up as equilibrium is approached and “self-retardation” of down jumps (*11*). The material-time formalism captures well this asymmetry (black dashed lines), and the measured data are predicted with a high accuracy for all down jumps and for up jumps up to 2 K. However, there is a clearly visible deviation for the largest (3 K) up jump, and in the Supplementary Materials it is documented that deviations in fact emerge already for a 2.5 K up jump. Thus, the formalism breaks down for large-amplitude up jumps. This may be related to only going to first order in the Taylor expansion in Eq. 8, but it could also be caused by the sample reaching equilibrium by other mechanisms than the one involved in smaller jumps. This may be similar to what is seen in the case of very large up jumps (30 to 70 K) performed on ultrastable vapor-deposited glasses where it has been shown that equilibrium is reached by heterogeneous growth of mobile domains (*54*). Alternatively, the deviations between data and predictions could be caused by beta processes playing a role in aging, as has been seen for polymers deep in the glass state (*55*, *56*).

The predictions agree well with the data of the nonlinear double jumps shown in Fig. 3B. This demonstrates that the material-time formalism works well also in this situation; we note that (*41*) presents an alternative approach for predicting the nonlinear aging response from linear data. The data shown in Fig. 3B are all from measurements in the temperature range where we have access to measured values of *X*_eq_ and to the fast contribution of *R*(*t*), while γ_eq_ used for the prediction is derived from an extrapolation of higher-temperature dielectric relaxation times. The parameter Λ is the same as for the single jumps, and the test of the nonlinear double-jump prediction is therefore performed with no free parameters. In contrast, in the classical Ritland-Kovacs crossover experiment (*46*, *48*, *49*), the first down jump goes deep into the glass state where the properties of the equilibrium liquid are not known. In the Supplementary Materials, we show data for a large down jump (7 K); the predictions using extrapolated parameters demonstrate qualitatively good results, although the formalism is not able to predict the time scale of aging in the temperature regime where equilibrium cannot be reached.

Last, Fig. 3C shows the response of the nonlinear sinusoidal temperature modulation along with the predictions. The lowest temperatures in the modulation are in a range where the parameters are extrapolated. Again, there are no free parameters in the prediction. The nonlinearity is seen as a sizable asymmetry in the peak shape: When the temperature is high, there is a substantial response, whereas the liquid responds much less to a decreased temperature. The gray horizontal dashed line corresponds to the equilibrium capacitance at the starting temperature 164.6 K. The asymmetry of the response is very well captured by the prediction. Because a large part of a sinusoidal is close to linear in time, and thus similar to a temperature ramp over several Kelvin, the aging in connection with a standard differential scanning calorimetry cooling or heating protocol is likewise expected to be predicted accurately. Deviations between prediction and data can be seen in the magnified panel of Fig. 3C and are most likely related to the first-order nature of Eq. 8.

The results in Fig. 3 demonstrate that nonlinear physical aging phenomena in the intermediate regime may be predicted from a knowledge of the linear limit of aging. Previous works have come close to this limit (*39*, *57*). While the linear limit is challenging to probe experimentally, it is conceptually important. First, it validates the central assumption of the material-time formalism. Second, linear response theory is well established via the fluctuation-dissipation (FD) theorem that predicts the response from thermal equilibrium fluctuations quantified via a time correlation function (*58*). Our results therefore imply that intermediate nonlinear physical aging can now, at least in principle and for relatively small jumps, be predicted from measurements of the equilibrium fluctuations, i.e., without perturbing the system at all. We end the paper by illustrating this possibility by presenting results from a computer simulation where thermal fluctuations are much easier to monitor than in experiments.

**Fig. 4.**
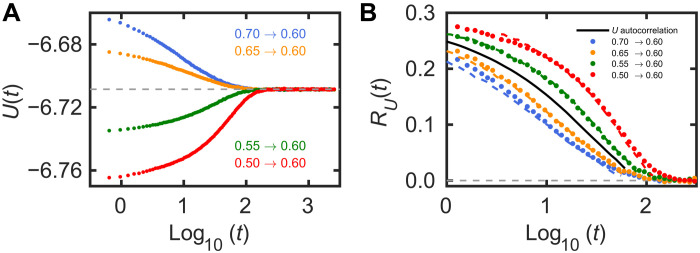
Results from computer simulations of a binary model liquid monitoring the potential energy. (**A**) Data for four temperature jumps to the same temperature (*T* = 0.60 in units based on the pair potential parameters). (**B**) The normalized relaxation function, *R_U_*(*t*), of the thermal equilibrium potential energy time autocorrelation function at this temperature (black line) and the predictions based on this for the temperature jumps (colored dashed curves). The data for the normalized relaxation functions based on (A) are shown as colored dots. The nonlinearity parameter was determined from the two smallest jumps in the same way as for the experimental data (see the Supplementary Materials).

The system studied is the binary Lennard-Jones (LJ) mixture of Kob and Andersen (*59*), which, for more than 20 years, has been the standard model for computer simulations of glass-forming liquids. We simulated a system of 8000 particles. The quantity monitored is the potential energy *U*. Temperature jump data were averaged over 1000 simulations to reduce the noise. Figure 4A shows results for jumps from four different temperatures to *T* = 0.60 (in simulation units), plotted as a function of the logarithm of the time passed after each jump was initiated. The curves are quite different, showing that the jumps are large enough to be notably nonlinear.

The FD theorem implies that the linear response to any small temperature variation is uniquely determined by the thermal equilibrium potential energy time autocorrelation function 〈*U*(0)*U*(*t*)〉 (*60*). We evaluated this quantity at *T* = 0.60. Using the single-parameter material-time formalism as above, we then predict nonlinear temperature jump results (Fig. 4B). The only free parameter is the Λ of Eq. 8, which is determined from the two smallest jumps (*53*). The colored dashed curves in Fig. 4B are the predictions for the normalized relaxation functions based on the black line in the middle that gives the thermal equilibrium normalized time autocorrelation function of the potential energy; the full circles are the normalized data from Fig. 4A. Overall, the predictions work well, demonstrating that intermediate nonlinear aging can be predicted from equilibrium fluctuations. The minor deviations for the two largest jumps are not unexpected, given that these involve temperature changes of more than 15% for which the single-parameter ansatz in Eq. 8 is likely not to be accurate.

## DISCUSSION

We have shown how physical aging involving temperature changes of a few percent can be predicted from the linear aging response, i.e., from the response to a very small temperature variation. This validates the central assumption of the material-time formalism. At the same time, it is clear that this formalism has limitations. Thus, the largest up jump (3 K) is not well predicted (Fig. 3A, bottom). This suggests that there are two regimes of nonlinear aging: an intermediate regime where the relaxation time varies for, at most, a few decades and the material-time concept describes the situation well, and a strongly nonlinear regime where the formalism breaks down and a new theoretical approach is needed. We speculate that even very large temperature down jumps may fall into the intermediate regime because the system here thermalizes gradually. This is in contrast to large up jumps, which are known to result in heterogeneous states very far from equilibrium (*54*). Aging far below the glass transition is also likely to deviate from the predictions because processes faster than the alpha relaxation may play a role here, particularly for polymers (*55*, *56*). Along this line of thinking, it is important to note that the standard glass transition resulting from a continuous cooling is likely to be described well by the material-time formalism, i.e., is intermediately nonlinear because vitrification for a constant cooling rate takes place over a narrow range of temperatures.

In regard to the intermediate aging regime, the implications of our findings are important both for the understanding of aging in application and for the theoretical interpretation of the aging dynamics. By reference to the FD theorem, the consequence is that the properties governing the intermediate nonlinear physical aging of a system far from equilibrium are embedded in the thermal equilibrium fluctuations and can be predicted from these. This means that there is no fundamental difference between the intermediate nonlinear and the linear aging responses. Understanding physical aging is therefore intimately linked to characterizing and understanding the spectral shapes of linear responses and autocorrelation functions, a classical field where there has been important recent progress both experimentally (*61*, *62*) and theoretically (*63*). The approach presented in this paper could prove useful for understanding the nonlinear response to electric fields. This is an active field (*64*–*66*) in which concepts from physical aging have been used successfully (*65*).

For future work, it would also be interesting to see how far the description of physical aging in terms of linear response can be extended by including higher-order terms in the Taylor expansion of Eq. 8. This can hopefully lead to a complete picture of which samples and protocols exhibit aging governed by the same processes as those responsible of the linear alpha relaxation and which situations involve other processes and mechanisms (*54*–*56*).

## MATERIALS AND METHODS

The study involves the glass-forming liquids VEC (99% purity) from Sigma-Aldrich for which data are shown in the main paper and NMEC (96% purity) from VWR for which data are shown in the Supplementary Materials. Both liquids were stored in a refrigerator at temperatures between 2° and 8°C and used as received.

For each liquid, a single sample was prepared for all the presented experiments. The sample cell was a plane-plate capacitor with a plate distance of 50 μm and a geometric capacitance of *C*_geo_ = 16 pF. The cell was filled under ambient conditions and immediately mounted into a precooled cryostat. VEC was quenched to *T*_cryo_ = 163 K, and NMEC was quenched to *T*_cryo_ = 167 K, at which the samples were kept to equilibrate for a couple of days. The temperature of the main cryostat was constant at *T*_cryo_ = 164 K for VEC and *T*_cryo_ = 167 K for NMEC during the experiments that lasted almost 1 year for each sample.

The temperature control of the experiments was obtained by a microregulator integrated with the capacitor sample cell. The regulation was achieved by controlling a Peltier element in contact with a capacitor plate. Temperature was monitored with a negative temperature coefficient resistor placed inside one of the capacitor plates. A figure showing the sample cell with a microregulator can be found in the Supplementary Materials. Further details on the microregulator and the main cryostat are given in (*37*). The microregulator can change temperature by steps of a few millikelvin up to several kelvin within seconds and keep the temperature constant with variations of less than 1 mK over weeks. All the temperature protocols shown, including the sinusoidal protocol, were achieved by making jumps in temperature with the microregulator.

The real and the imaginary part of the capacitance at 10 kHz was monitored during the entire experiment with a sampling rate of approximately one measurement per second. The measurements were performed using an AH2700A Andeen Hagerling ultraprecision capacitance bridge. It is the combination of the fast and precise temperature control with the high precision of the bridge that makes it possible to measure aging in the linear limit.

The simulations used the Kob-Andersen 80/20 binary LJ mixture (*59*), which was simulated by means of standard *NVT* Nosé-Hoover dynamics (*67*) using the GPU-optimized software RUMD (*68*). A system of 8000 particles was simulated. In LJ units, the time step was 0.0025. All pair potentials were cut and shifted at 2.5 times the length parameter σ*_ij_* of the relevant LJ pair potential (*i*, *j* = *A*, *B*). At the reference temperature *T* = 0.60, the potential energy time autocorrelation function was calculated as follows: First, 10^7^ time steps of simulations were carried out for equilibration. After that, the time autocorrelation function was calculated using the fast Fourier transform. The temperature jump simulations were carried out by the following procedure applied for all starting temperatures. First, 5 × 10^5^ time steps were spent on equilibration at the given starting temperature. After that, a total of 5 × 10^8^ time steps were spent on the production runs from which 1000 independent configurations were selected to serve as starting configurations for a temperature jump to *T* = 0.60. The Fig. 4 data represent averages over these 1000 jumps.

## Supplementary Materials

### This PDF file includes:

Supplementary Information A to C Figs. S1 to S27 Tables S1 and S2 References
