# Supplementary material for: Predicting nonlinear physical aging of glasses from equilibrium relaxation via the material time
Source: Sci Adv. 2022 Mar 16;8(11):eabl9809. doi: 10.1126/sciadv.abl9809 (PMC8926348; doi:10.1126/sciadv.abl9809)
Supplement: Supplementary file 1 — Supplementary Information A to C Figs. S1 to S27 Tables S1 and S2 References [file sciadv.abl9809_sm.pdf]

Science Advances
AAAS

Supplementary Materials for

Predicting nonlinear physical aging of glasses from equilibrium relaxation via the material time

Birte Riechers, Lisa A. Roed, Saeed Mehri, Trond S. Ingebrigtsen, Tina Hecksher, Jeppe C. Dyre*, Kristine Niss*

*Corresponding author. Email: dyre@ruc.dk (J.C.D.); kniss@ruc.dk (K.N.)

Published 16 March 2022, *Sci. Adv.* **8**, eabl9809 (2022)
DOI: 10.1126/sciadv.abl9809

This PDF file includes:

Supplementary Information A to C
Figs. S1 to S27
Tables S1 and S2
References

Supplementary Note 1: Electronic structure – local density of states

The local density of states of the considered systems as is shown in Supplementary Figure S1.

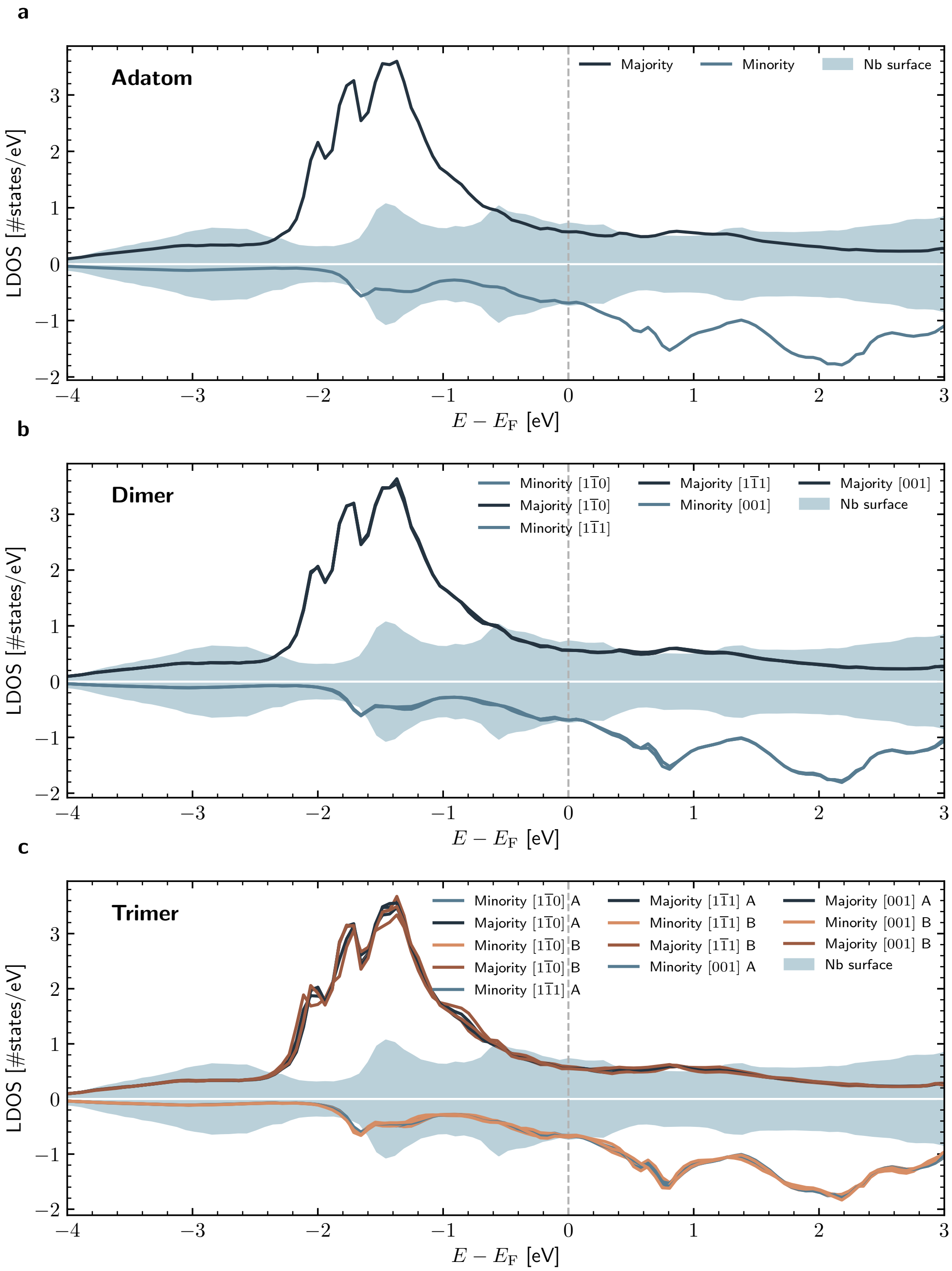

Figure S1: Local density of states (LDOS) of the different considered systems. The filled background shows the LDOS of the first Nb surface layer, while the lines show the LDOS of the adatom (**a**), the dimers (**b**), and the trimers (**c**).

Supplementary Note 2: Fano shape fit of the zero-bias anomaly

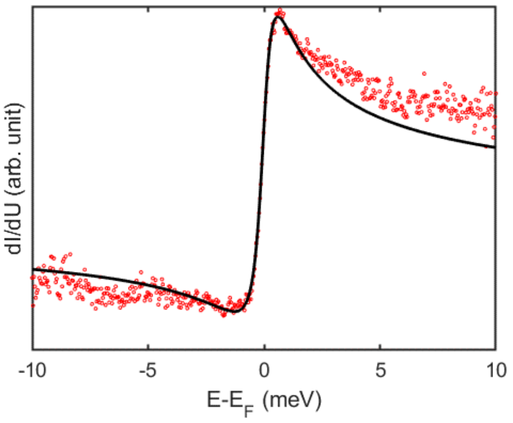

$$F(E) = F_0 + A \cdot Re\left(e^{i\varphi} \sqrt{\frac{i\Gamma/2}{E - E_F + i\Gamma/2}} \right)$$

Γ = 0.97meV

φ = -1.33

$$T_K = \frac{\Gamma}{1.455 k_B} = \mathbf{7.74K}$$

Figure S2: Fit of the zero-bias anomaly for a single Cr adatom according to a Frota function. The red dots correspond to the experimental spectrum while the black line corresponds to the fit. To minimize the influence of the tip density of states, the spectrum has been normalized to the one acquired by positioning the tip over the bare Nb(110) substrate using the very same W microtip. Measurements have been acquired under a magnetic field of $B = 2$ T applied perpendicular to the sample surface to drive the Nb substrate into the metallic regime.

Supplementary Note 3: Measurements at different set-points currents

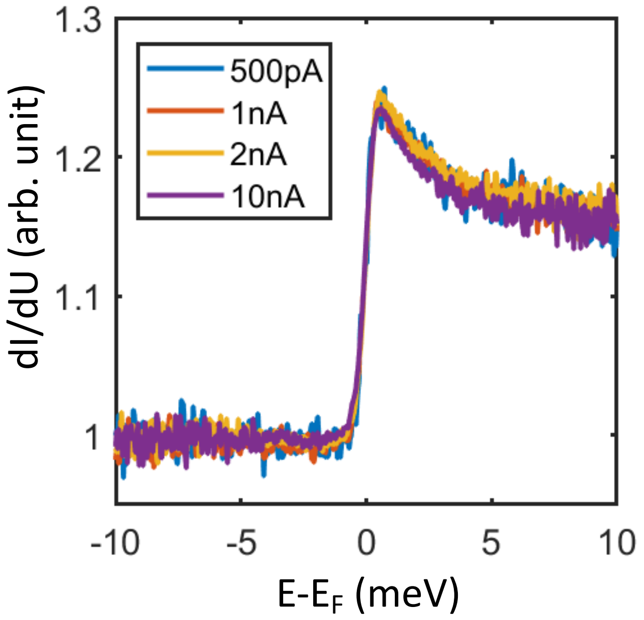

Figure S3: Zero-bias anomaly for a single Cr adatom acquired by stabilizing the tip at progressively higher tunneling currents. The zero bias anomaly does not show any spectral change by increasing the current.

Supplementary Note 4: Spatial mapping of the zero-bias anomaly

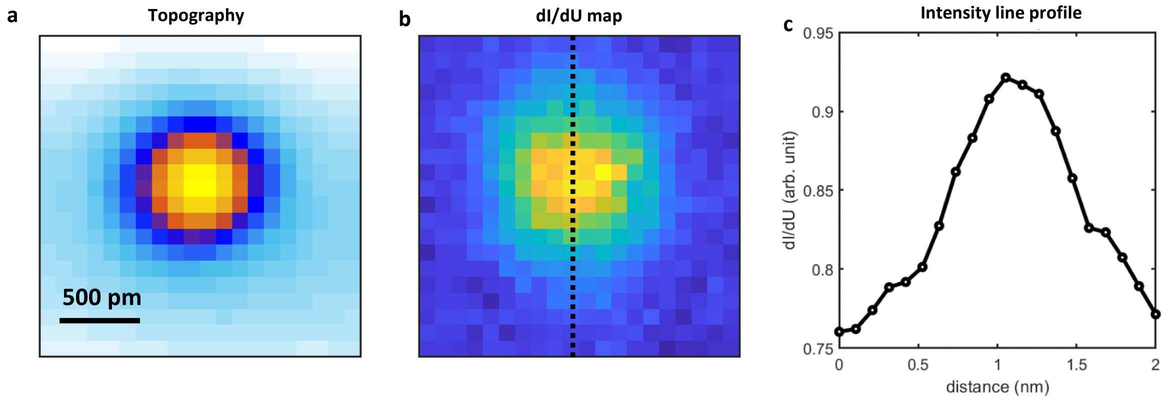

Figure S4: Spatial mapping of the zero-bias anomaly of Cr adatom deposited on the Nb(110) surface. **a** Topography of the probed single Cr adatom. **b** Spatial mapping of the zero-bias anomaly acquired by a full spectroscopic grid. The map corresponds to the intensity of the step-like spectroscopic signature at the energy E = 0.750 μV. **c** Corresponding line profile of the intensity along the black dashed line in **b**. The data allow to visualize that the zero-bias anomaly is strongly localized onto the Cr adatom.

Supplementary Note 5: Zero bias anomaly on Mn

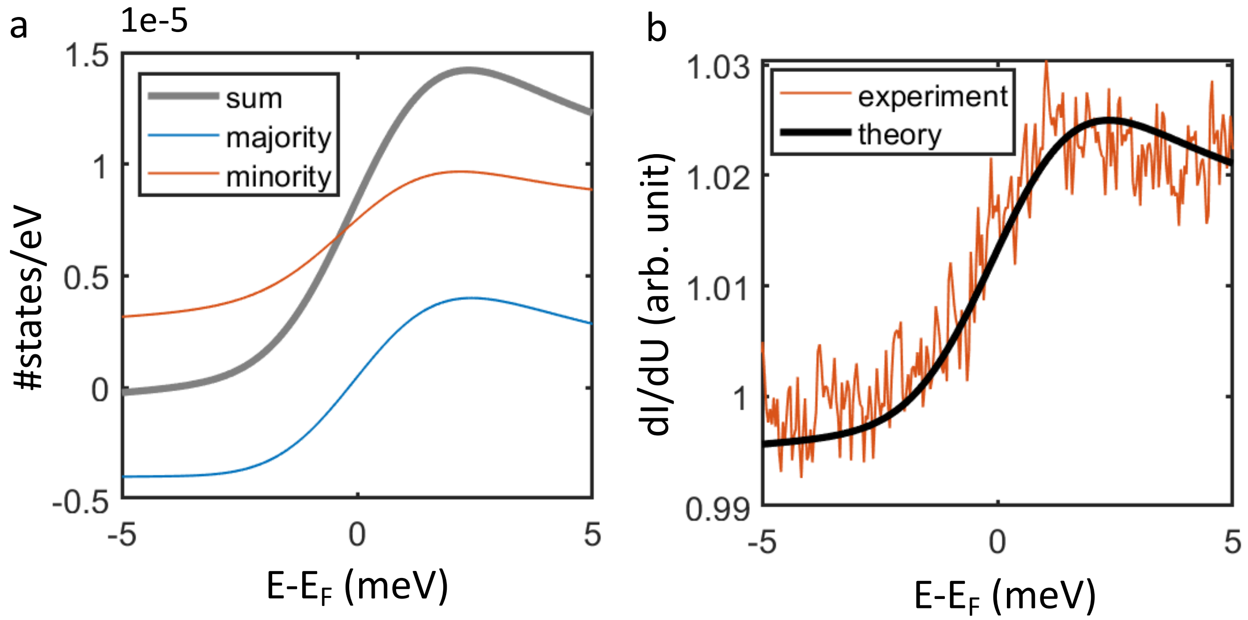

Figure S5: Zero-bias anomaly for a single Mn adatom coupled to the Nb(110) surface. Similarly to Cr, a step-like spectroscopic signature centered at the Fermi level is found also for Mn as obtained theoretically from ab-initio **a** and experimentally **b**. Compared to Cr, the Mn signature is broader and weaker in intensity. As evident by comparing panels **a** and **b**, also for Mn the theoretically calculated spectra can describe the experimental results with high accuracy.

Supplementary Note 6: Symmetry analysis of the magnetic interactions

To analyse the ab-initio results, we use throughout this paper a generalized Heisenberg model,

$$\mathcal{H} = \sum_i \mathbf{e}_i \mathcal{K}_i \mathbf{e}_i + \frac{1}{2}\sum_{ij} J_{ij}\mathbf{e}_i \cdot \mathbf{e}_j + \frac{1}{2}\sum_{ij} \mathbf{D}_{ij} \cdot \mathbf{e}_i \times \mathbf{e}_j + \frac{1}{2}\sum_{ij} \mathbf{e}_i J_{ij}^{\mathrm{sym}} \mathbf{e}_j - \sum_i m_i \mathbf{B}_i \cdot \mathbf{e}_i \quad , \tag{S.1}$$

where i labels the Cr atoms with $\mathbf{e}_i$ being the direction of its magnetic moment, $\mathcal{K}_i$ is the magnetic on-site anisotropy, J_{ij} is the isotropic exchange interaction, $\mathbf{D}_{ij}$ is the Dzyaloshinskii-Moriya interaction, J_{ij}^{sym} is the symmetric anisotropic exchange interaction, and $\mathbf{B}_i$ is an external magnetic field. Depending on the symmetry of the system the allowed form of the interactions can vary.

Adatom in C_{2v} symmetry. The isolated adatom follows the C_{2v} symmetry of the underlying Nb(110) lattice, which results in a magnetic anisotropy matrix of the following form:

$$\mathcal{K} = \begin{pmatrix} \mathcal{K}_x & 0 & 0 \\ 0 & \mathcal{K}_y & 0 \\ 0 & 0 & 0 \end{pmatrix} \quad . \tag{S.2}$$

Note that the zz-component was set to 0, which results from the freedom of the trace of the magnetic anisotropy.

Dimer in C_{2v} and C_2 symmetry. The Cr dimers oriented in the high-symmetry directions [001] and [1$\bar{1}$0] preserve the C_{2v} symmetry of the underlying lattice, while the dimer in the [1$\bar{1}$1] has the reduced symmetry C_2. Even though the magnetic on-site anisotropy is in general complex with off-diagonal elements (due to the lack of local symmetries, at most one mirror plane is present for the discussed dimers), we assume it to be similar to the one of the isolated adatom since we discuss only next-nearest neighbor dimers resulting in a weak coupling and only a weak renormalization of the on-site anisotropy is expected. This assumption is supported by the band energy differences in the frozen potential approximation shown in Table 1 of the main text, which do not show any siginificant renormalizations of the anisotropy when dimers and trimers are built.
In the general case only a rotation along the z axis by 180° is present leading to the symmetry constraints,

$$\mathbf{D}_{12} = (D_x\,,\, D_y\,,\, 0) \qquad \text{and} \qquad J_{12}^{\mathrm{sym}} = \begin{pmatrix} J_{xx} & J_{xy} & 0 \\ J_{xy} & J_{yy} & 0 \\ 0 & 0 & -J_{xx} - J_{yy} \end{pmatrix} \quad , \tag{S.3}$$

while $J_{xy} = 0$, and $D_y = 0$ and $D_x = 0$, respectively, for the dimers in the high-symmetry directions [001] and [1$\bar{1}$0].

Trimer in the C_{2v} and C_2 symmetry. Similarly to the dimers the trimers can be grouped into the trimers preserving the C_{2v} symmetry of the lattice (those along high-symmetry directions) and the [1$\bar{1}$1] trimer which reduces the symmetry to C_2. The magnetic on-site anisotropy is assumed to be similar to the one of the isolated adatom. For the trimers there are two possible types of interactions connecting either direct neighbors (atoms 1 and 2 and atoms 2 and 3) or the end atoms of the trimer (atoms 1 and 3). The interactions of the former pairs can always be related to each other using the rotational symmetry along the z-axis present in C_2 and C_{2v}. These interactions simplify further for the trimers along the high-symmetry directions leading D_y and D_x as the only finite off-diagonal components for the [001] and the [1$\bar{1}$0] direction, respectively. The interaction between the two end atoms of each trimer follows the same symmetry rules as the one of the dimers in the respective directions.

Supplementary Note 7: Magnetic ground states

The magnetic ground states of the systems can be either obtained by minimizing the Heisenberg model, eq. (S.1), with parameters obtained from DFT (see Methods of the main text, Table 1 of the main text, and Supplementary Table **S1**), or by calculating the non-collinear ground state self-consistently from DFT. The latter approach is computationally more expensive, but also more precise, since it includes for example potential higher-order contributions to the magnetic anisotropy or the magnetic exchange interactions (see e.g. Ref. [69]). We used this approach to obtain the magnetic ground states of all systems, which are shown in Supplementary Table **S2**.

Adatom. The magnetic moment in the zero field ground state of the adatom is parallel to the y-direction, which is favoured by the magnetic on-site anisotropy,

$$\mathcal{K} = \begin{pmatrix} -0.188 & 0.0 & 0.0 \\ 0.0 & -0.200 & 0.0 \\ 0.0 & 0.0 & 0.0 \end{pmatrix} \mathrm{meV} \quad . \tag{S.4}$$

When a field is applied in the out-of-plane direction (z-direction), our self-consistent first-principles calculations indicate that the adatom starts to tilt towards the out-of-plane direction, and for fields $\geq 3\,\mathrm{T}$ it fully aligns with the field.

Dimer. The ferromagnetic dimer in the $[1\bar{1}1]$ direction almost aligns with the magnetic field exhibiting a small tilt of 2.4° in the polar angles due to the DMI (see Supplementary Table **S1**). The other dimers stay mostly in their AFM in-plane ground state with only a weak impact of the field. Noteworthy, the [001] dimer even tilts towards the $-z$-direction counteracting the field, which is due to a competition with the relatively strong DMI in the system.

Trimer. The ferromagnetic $[1\bar{1}1]$ trimer aligns with the field with a small opening of 4.3°. Compared to the previously discussed antiferromagnetic dimers, the antiferromagnetic trimers show a quite different behaviour. Due to the odd number of atoms, the trimers exhibit a finite net magnetic moment, and can therefore respond to a magnetic field without competing with the internal strong AFM exchange. If we ignore the internal spin structrue, they act like a macrospin with the same magnetic moment as a single adatom but three times larger magnetic anistropy. Therefore, the weak AFM trimer in the $[1\bar{1}0]$ direction tilts towards the out-of-plane direction with an effective polar angle of 64.7°. The strong AFM trimer in the [001] direction shows additional cantings induced by a strong DMI.

	$Cr_2[1\bar{1}0]$	$Cr_2[001]$	$Cr_2[1\bar{1}1]$	$Cr_3[1\bar{1}0]$	$Cr_3[001]$	$Cr_3[1\bar{1}1]$
$\lvert\mathbf{D}_1\rvert$ [meV]	0.05 meV	0.21 meV	0.84 meV	0.05 meV	0.33 meV	0.81 meV

Table S1: Magnitude of the next nearest-neighbor Dzyaloshinskii-Moriya interaction. The magnetic interaction $\lvert\mathbf{D}_1\rvert$ among the closest neighbors is stable with the size of the nanostructures (Cr atoms are not neareast neighbors) but changes dramatically with their crystallographic direction.

System	Polar angles (scf)	Azimuthal angles (scf)
Adatom (0 T)	90°	90°
Adatom (1 T)	64°	90°
Adatom (2 T)	30°	90°
Adatom (≥3 T)	0°	0°
$[1\bar{1}1]$ dimer (5 T)	(2.4°, 2.4°)	(−66.5°, 113.5°)
$[1\bar{1}0]$ dimer (5 T)	(87.5°, 87.5°)	(90°, −90°)
[001] dimer (5 T)	(91.3°, 91.3°)	(90°, −90°)
$[1\bar{1}1]$ trimer (5 T)	(4.3°, 0.0°, 4.3°)	(−72.5°, −101.9°, 107.5°)
$[1\bar{1}0]$ trimer (5 T)	(64.7°, 113.5°, 64.7°)	(89.6°, −90.0°, 90.4°)
[001] trimer (5 T)	(86.4°, 98.3°, 76.0°)	(90.0°, −90.0°, 90.0°)

Table S2: Magnetic ground state of the adatom, dimers and trimers on the Nb(110) surface obtained self-consistently from DFT. The polar and azimuthal angles define the direction of the magnetic moments carried by the adatoms.

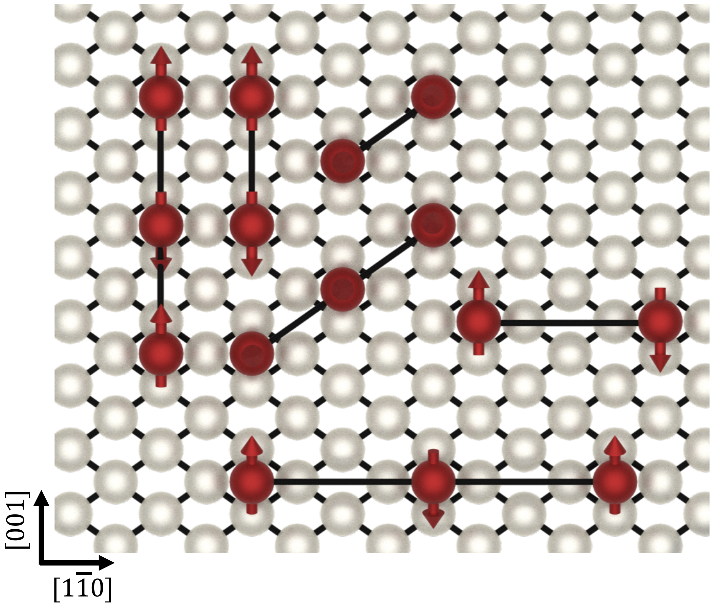

Figure S6: Illustration of the magnetic ground states of all dimers and trimers on the Nb(110) surface. An out-of-plane magnetic field of 5 T is assumed. The Cr nanostructures are represented by red spheres with arrows connected by black lines while the Nb atoms are shown as grey spheres. The corresponding polar and azimuthal angles are given in Supplementary Table S2.

Supplementary Note 8: Landau-Lifshitz-Gilbert model

The Landau-Lifshitz-Gilbert (LLG) model [55,56] describes the transversal dynamics of magnetic moments, and can in general be written as

$$\frac{\mathrm{d}\mathbf{m}_i}{\mathrm{d}t} = -\gamma \mathbf{m}_i \times \left(\mathbf{B}_i^{\mathrm{eff}} + \sum_j \mathcal{G}_{ij} \cdot \frac{\mathrm{d}\mathbf{m}_j}{\mathrm{d}t} \right) \quad , \tag{S.5}$$

where γ is the gyromagnetic ratio, $\mathbf{B}_i^{\mathrm{eff}}$ is the effective field acting on the moment i and $\mathcal{G}_{ij}$ is the Gilbert damping tensor. The effective field can be obtained using any convenient spin model like the generalized Heisenberg model discussed in Supplementary Note 6:. It can be shown that in the LLG model the transversal magnetic susceptibility can be calculated by

$$\chi^{-1}_{i\alpha j\beta}(\omega) = \delta_{ij}\left(\delta_{\alpha\beta}\frac{B_{iz}^{\mathrm{eff}}}{M_i} + \frac{\mathrm{i}\,\omega}{\gamma M_i}\epsilon_{\alpha\beta\mu}\right) + \frac{1}{M_i M_j}(\mathcal{R}_i J_{ij} \mathcal{R}_j^{\mathsf{T}})_{\alpha\beta} + \mathrm{i}\,\omega(\mathcal{R}_i \mathcal{G}_{ij} \mathcal{R}_j^{\mathsf{T}})_{\alpha\beta} \quad , \tag{S.6}$$

where $\mathcal{R}_i$ describes a rotation to the local spin frame of the magnetic moment i, which is oriented such that the local z-coordinate aligns with the direction of the magnetic moment. In the following, we will approximate the Gilbert damping tensor by $\mathcal{G}_{ij} = \delta_{ij}\alpha/\gamma m_i$, where α is the isotropic Gilbert damping parameter. The equally important off-diagonal elements of the Gilbert damping tensor can be shown to renormalize the gyromagnetic ratio and can thus be neglected if we assume a general gyromagnetic ratio with $\gamma \neq 2$.

Adatoms in a magnetic field The first example we discuss is the single adatom in an external magnetic field. We assume an out-of-plane orientation of the magnetic moment of the adatom, which holds for our system for fields $\gtrsim 2\,\mathrm{T}$ (see Supplementary Note 7:). Using the spin model of the single adatom discussed in eqs. (S.1) and (S.2) and the definition of the inverse transverse susceptiblity in the LLG model given in eq. (S.6) one finds,

$$\chi^{-1}(\omega) = \begin{pmatrix} \frac{K_x}{M^2} + \frac{B}{M} + \mathrm{i}\frac{\alpha\omega}{\gamma M} & \mathrm{i}\frac{\omega}{\gamma M} \\ -\mathrm{i}\frac{\omega}{\gamma M} & \frac{K_y}{M^2} + \frac{B}{M} + \mathrm{i}\frac{\alpha\omega}{\gamma M} \end{pmatrix} \quad , \tag{S.7}$$

with the eigenfrequencies given by $\det(\chi^{-1}(\omega)) = 0$ leading to,

$$\omega_\pm = \frac{\gamma}{(1+\alpha^2)}\frac{1}{2M}\left(\mathrm{i}\alpha\,(K_x + K_y + 2BM) \pm \sqrt{4(K_x+BM)(K_y+BM) - \alpha^2(K_x-K_y)^2}\right) \quad . \tag{S.8}$$

Defining $\gamma' = \frac{\gamma}{1+\alpha^2}$ the broadening (defining the FWHM of the exciations) of the eigenmodes is given by

$$\mathrm{Im}(\omega_\pm) = \alpha\gamma'\frac{K_x + K_y + 2BM}{2M} \quad , \tag{S.9}$$

while the energies of the eigenmodes are given by,

$$\mathrm{Re}(\omega_\pm) = \pm\frac{\gamma'}{2M}\sqrt{4(K_x+BM)(K_y+BM) - \alpha^2(K_x-K_y)^2} \quad . \tag{S.10}$$

Note that in the main manuscript we simplify the equations by using $K_x = K_y$, which is a very good approximation for the discussed Cr adatom (see eq. S.4), and consequently also for the dimers and trimers.

Collinear dimers Considering only on-site anisotropies and isotropic exchange interactions, the effective fields are given by

$$\mathbf{B}_i^{\mathrm{FM/AFM}} = \mp\frac{\mathbf{e}_z}{M}J \quad , \quad i = \{1,2\} \quad . \tag{S.11}$$

Assuming a local and isotropic Gilbert damping tensor, $\mathcal{G}_{ij,\alpha\beta} = \delta_{ij}\delta_{\alpha\beta}\alpha$, one finds for the susceptibility of the ferromagnetic dimer in the $\{x, y\}$-basis,

$$\chi_{\mathrm{FM}}^{-1} = \begin{pmatrix} \frac{-J+K_x}{M^2} + \frac{\mathrm{i}\alpha\omega}{\gamma M} & \mathrm{i}\frac{\omega}{\gamma M} & J & 0 \\ -\mathrm{i}\frac{\omega}{\gamma M} & \frac{-J+K_y}{M^2} + \frac{\mathrm{i}\alpha\omega}{\gamma M} & 0 & J \\ J & 0 & \frac{-J+K_x}{M^2} + \frac{\mathrm{i}\alpha\omega}{\gamma M} & \mathrm{i}\frac{\omega}{\gamma M} \\ 0 & J & -\mathrm{i}\frac{\omega}{\gamma M} & \frac{-J+K_y}{M^2} + \frac{\mathrm{i}\alpha\omega}{\gamma M} \end{pmatrix} \tag{S.12}$$

with the poles given by $\det(\chi^{-1}(\omega)) = 0$ yielding,

$$\begin{aligned} \omega_{\mathrm{ac}}^{\pm} &= \frac{\gamma}{2M\left(1+\alpha^2\gamma^2\right)}\left[\mathrm{i}\alpha\gamma(K_x+K_y) \pm \sqrt{4K_xK_y - \alpha^2\gamma^2(K_x-K_y)^2}\right] \\ \omega_{\mathrm{op}}^{\pm} &= \frac{\gamma}{2M\left(1+\alpha^2\gamma^2\right)}\left[\mathrm{i}\alpha\gamma(-4J+K_x+K_y) \pm \sqrt{(4J-(K_x+K_y))^2-(K_x-K_y)^2-\alpha^2\gamma^2(K_x-K_y)^2}\right] \quad . \end{aligned} \tag{S.13}$$

For antiferromagnetic dimers the different local spin frames and the different effective magnetic field lead to

$$\chi_{\mathrm{AFM}}^{-1} = \begin{pmatrix} \frac{J+K_x}{M^2} + \frac{\mathrm{i}\alpha\omega}{\gamma M} & \mathrm{i}\frac{\omega}{\gamma M} & -J & 0 \\ -\mathrm{i}\frac{\omega}{\gamma M} & \frac{J+K_y}{M^2} + \frac{\mathrm{i}\alpha\omega}{\gamma M} & 0 & J \\ -J & 0 & \frac{J+K_x}{M^2} + \frac{\mathrm{i}\alpha\omega}{\gamma M} & \mathrm{i}\frac{\omega}{\gamma M} \\ 0 & J & -\mathrm{i}\frac{\omega}{\gamma M} & \frac{J+K_y}{M^2} + \frac{\mathrm{i}\alpha\omega}{\gamma M} \end{pmatrix} \quad , \tag{S.14}$$

with poles at

$$\begin{aligned} \omega_{\mathrm{op},1}^{\pm} &= \frac{\gamma}{2M\left(1+\alpha^2\gamma^2\right)}\left[\mathrm{i}\alpha\gamma(2J+K_x+K_y) \pm \sqrt{4K_x(2J+K_y) - \alpha^2\gamma^2(2J-K_x+K_y)^2}\right] \\ \omega_{\mathrm{op},2}^{\pm} &= \frac{\gamma}{2M\left(1+\alpha^2\gamma^2\right)}\left[\mathrm{i}\alpha\gamma(2J+K_x+K_y) \pm \sqrt{4K_y(2J+K_x) - \alpha^2\gamma^2(2J+K_x-K_y)^2}\right] \quad . \end{aligned} \tag{S.15}$$

Note that the parametrization of the discussed LLG model holds for collinear dimers (FM or AFM) with out-of-plane magnetic moments, and small deviations are expected when using the realistic magnetic ground states of the dimers presented in Supplementary Note 7:.

Collinear trimers Similar to the dimers we consider only on-site anisotropies and isotropic exchange interactions, which yields for the effective fields in the ferromagnetic case,

$$\mathbf{B}_i^{\mathrm{FM}} = \frac{\mathbf{e}_z}{M}\begin{cases} -J_1 - J_2 & , \quad i = \{1,3\} \\ -2J_1 & , \quad i = 2 \end{cases} \quad , \tag{S.16}$$

giving rise to the susceptibility in the $\{x, y\}$-basis,

$$\chi_{\mathrm{FM}}^{-1} = \begin{pmatrix} \frac{-J_1-J_2+K_x}{M^2} + \frac{\mathrm{i}\alpha\omega}{\gamma M} & \mathrm{i}\frac{\omega}{\gamma M} & J_1 & 0 & J_2 & 0 \\ -\mathrm{i}\frac{\omega}{\gamma M} & \frac{-J_1-J_2+K_y}{M^2} + \frac{\mathrm{i}\alpha\omega}{\gamma M} & 0 & J_1 & 0 & J_2 \\ J_1 & 0 & \frac{-2J_1+K_x}{M^2} + \frac{\mathrm{i}\alpha\omega}{\gamma M} & \mathrm{i}\frac{\omega}{\gamma M} & J_1 & 0 \\ 0 & J_1 & -\mathrm{i}\frac{\omega}{\gamma M} & \frac{-2J_1+K_y}{M^2} + \frac{\mathrm{i}\alpha\omega}{\gamma M} & 0 & J_1 \\ J_2 & 0 & J_1 & 0 & \frac{-J_1-J_2+K_x}{M^2} + \frac{\mathrm{i}\alpha\omega}{\gamma M} & \mathrm{i}\frac{\omega}{\gamma M} \\ 0 & J_2 & 0 & J_1 & -\mathrm{i}\frac{\omega}{\gamma M} & \frac{-J_1-J_2+K_y}{M^2} + \frac{\mathrm{i}\alpha\omega}{\gamma M} \end{pmatrix} \tag{S.17}$$

with the poles given by $\det(\chi^{-1}(\omega)) = 0$ yielding,

$$\omega_1^\pm = \frac{\gamma}{2M\left(1+\alpha^2\gamma^2\right)}\left[\mathrm{i}\alpha\gamma(K_x+K_y) \pm \sqrt{4K_xK_y - \alpha^2\gamma^2(K_x-K_y)^2}\right]$$
$$\omega_2^\pm = \frac{\gamma}{2M\left(1+\alpha^2\gamma^2\right)}\left[\mathrm{i}\alpha\gamma(-6J_1+K_x+K_y) \pm \sqrt{(6J_1-(K_x+K_y))^2-(K_x-K_y)^2-\alpha^2\gamma^2(K_x-K_y)^2}\right]$$
$$\omega_3^\pm = \frac{\gamma}{2M\left(1+\alpha^2\gamma^2\right)}\Bigg[\mathrm{i}\alpha\gamma(-2J_1-4J_2+K_x+K_y)$$
$$\pm\sqrt{(2J_1+4J_2)^2+4K_xK_y-(4J_1+8J_2)(K_x+K_y)-\alpha^2\gamma^2(K_x-K_y)^2}\Bigg] \quad . \tag{S.18}$$

For the antiferromagnetic trimer (central atom pointing along $\mathbf{e}_2 = -\mathbf{e}_z$) the effective magnetic field is given by

$$\mathbf{B}_i^{\mathrm{AFM}} = \frac{\mathbf{e}_z}{M}\begin{cases} J_1 - J_2 & , \quad i = \{1,3\} \\ 2J_1 & , \quad i = 2 \end{cases} \tag{S.19}$$

leading to the susceptibility represented in the $\{x,y\}$-basis by

$$\chi_{\mathrm{AFM}}^{-1} = \begin{pmatrix} \frac{J_1-J_2+K_x}{M^2}+\mathrm{i}\alpha\omega & \mathrm{i}\frac{\omega}{\gamma M} & -J_1 & 0 & J_2 & 0 \\ -\mathrm{i}\frac{\omega}{\gamma M} & \frac{J_1-J_2+K_y}{M^2}+\mathrm{i}\alpha\omega & 0 & J_1 & 0 & J_2 \\ -J_1 & 0 & \frac{2J_1+K_x}{M^2}+\mathrm{i}\alpha\omega & \mathrm{i}\frac{\omega}{\gamma M} & -J_1 & 0 \\ 0 & J_1 & -\mathrm{i}\frac{\omega}{\gamma M} & \frac{2J_1+K_y}{M^2}+\mathrm{i}\alpha\omega & 0 & J_1 \\ J_2 & 0 & -J_1 & 0 & \frac{J_1-J_2+K_x}{M^2}+\mathrm{i}\alpha\omega & \mathrm{i}\frac{\omega}{\gamma M} \\ 0 & J_2 & 0 & J_1 & -\mathrm{i}\frac{\omega}{\gamma M} & \frac{J_1-J_2+K_y}{M^2}+\mathrm{i}\alpha\omega \end{pmatrix} \tag{S.20}$$

The poles cannot be brought into a neat form as shown for the FM case in eq. (S.18), but we can discuss the limit of an uniaxial anisotropy with $K_y = K_x$, which yields for the eigenfrequencies,

$$\omega_{\mathrm{ac}}^\pm = \pm\frac{1}{2M}\frac{\sqrt{-\gamma^2\left(J_1^2(3\alpha\gamma+\mathrm{i})^2+4\mathrm{i}J_1K_x(\alpha\gamma+3\mathrm{i})-4K_x^2\right)}+\mathrm{i}\alpha\gamma^2(3J_1+2K_x)-\gamma J_1}{\alpha^2\gamma^2+1} \tag{S.21}$$
$$\omega_{\mathrm{op},1}^\pm = \frac{1}{M}\frac{\mathrm{i}\gamma(J_1-2J_2+K_x)}{\alpha\gamma\pm\mathrm{i}} \tag{S.22}$$
$$\omega_{\mathrm{op},2}^\pm = \frac{1}{2M}\frac{\pm\sqrt{-\gamma^2\left(J_1^2(-3\alpha\gamma+\mathrm{i})^2+4J_1K_x(-3-\mathrm{i}\alpha\gamma)-4K_x^2\right)}+\mathrm{i}\alpha\gamma^2(3J_1+2K_x)+\gamma J_1}{\alpha^2\gamma^2+1} \quad . \tag{S.23}$$

The eigenfrequencies $\omega_{\mathrm{op},1}$ and $\omega_{\mathrm{op},2}$ are at high energies mainly driven by the exchange J_1, while ω_2 yields a modes which is mainly driven by the on-site anisotropy K_x. To get more insights in the latter one, we can expand ω_{ac} further using the limits $\alpha \to 0$ and $J_1 \gg K_x$ yielding, $\omega_2^\pm = \pm 3\gamma K_x$, which is three times larger than the acoustical mode of the ferromagnetic trimer, eq. (S.18), and the corresponding mode of the isolated atom, eq. (S.10). Solving the generalized eigenvalue problem, $\chi^{-1}(\omega)\delta\mathbf{M} = 0$, yields the nature of this mode, which is in the global spin frame given by

$$\delta\mathbf{M}_{\mathrm{ac}}^\pm = (\pm\mathrm{i}, 1, \mp\mathrm{i}, -1, \pm\mathrm{i}, 1) \quad , \tag{S.24}$$

describing the collinear motion of the three anti-ferromagnetically coupled trimer atoms.
Note that this behaviour is fundamentally different from the AFM dimer, which only exhibits optical modes at energies related to the magnetic exchange J_1, see eq. (S.15). The main reason for this distinct response is that the AFM trimer has a net magnetic moment which can respond to an external magnetic field. This net magnetic moment, however, is similar to the one of the isolated atoms magnetic moment resulting in a moment per atom reduced by a factor of 3, which in turn gives rise to the enhanced excitation energy of the acoustical mode.

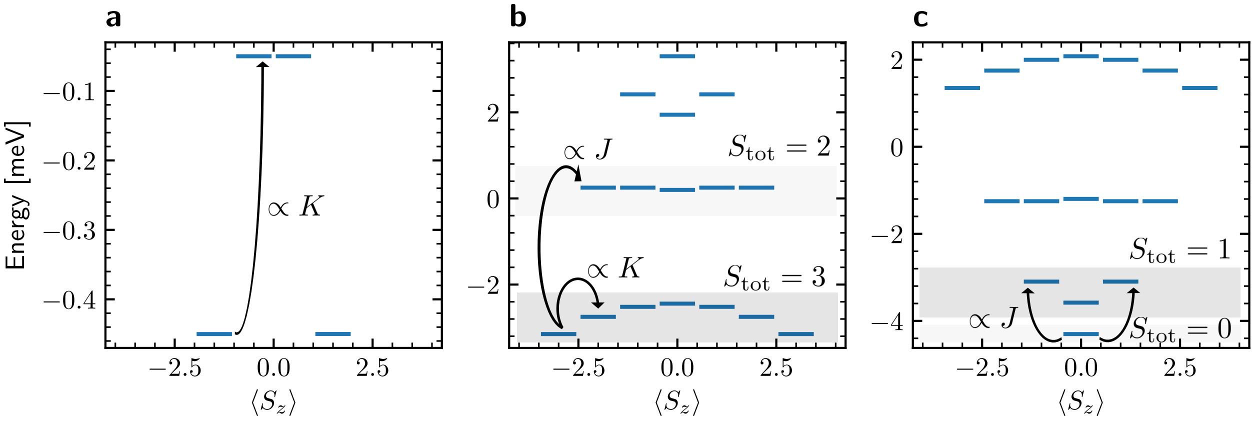

Figure S7: Level diagrams of an isolated 3/2-spin and two magnetically coupled 3/2-spins. a Isolated spin with a uniaxial anisotropy, $K = 0.2\,\mathrm{meV}$. **b** Ferromagnetically coupled spins with $J = -1\,\mathrm{meV}$ and $K_1 = K_2 = 0.2\,\mathrm{meV}$. **c** Antiferromagnetically coupled spins with $J = 1\,\mathrm{meV}$ and $K_1 = K_2 = 0.2\,\mathrm{meV}$.

Supplementary Note 9: Excitions in the quantum spin picture

Instead of the semi-classical picture which we employed in this work, magnetic atoms on surfaces are often described using the quantum spin picture. Note that on a metal due to strong hybridizations of the spin states with the surface, the ideal quantum picture of sharp isolated levels is not valid, but broadenings would have to be included. Here, we want to give an intuitive argument for the absence of an acoustical mode in an antiferromagnetically coupled dimer. Supplementary Figure S7a shows the level diagram of an 3/2-spin with uniaxial anisotropy, $H = K\sqrt{S(S+1)}\,\hat{S}_z^2$. This quantum spin system can be excited from the $m = -3/2$ state to the $m = -1/2$ state with a transition energy proportional to the anisotropy. The case of a ferromagnetically coupled dimer composed out of two 3/2-spins which are coupled by an isotropic magnetic exchange with $J \gg K$ is shown in Supplementary Figure S7b. The acoustical mode corresponds to an excitation within the subspace $S_{\rm tot} = 3$, which is proportional to the magnetic anisotropy. The optical mode, however, corresponds to an excitation from the $S_{\rm tot} = 3$ to the $S_{\rm tot} = 2$ subspace, which is proportional to the magnetic exchange J. In contrast, the antiferromagnetically coupled dimer, which is shown in Supplementary Figure S7c, does not exhibit an accoustical mode since its ground state is the $S_{\rm tot} = 0$ subspace. Thus, only the optical mode is present.

Supplementary Note 10: Comparison of raw data for substrate, single atoms, and dimer

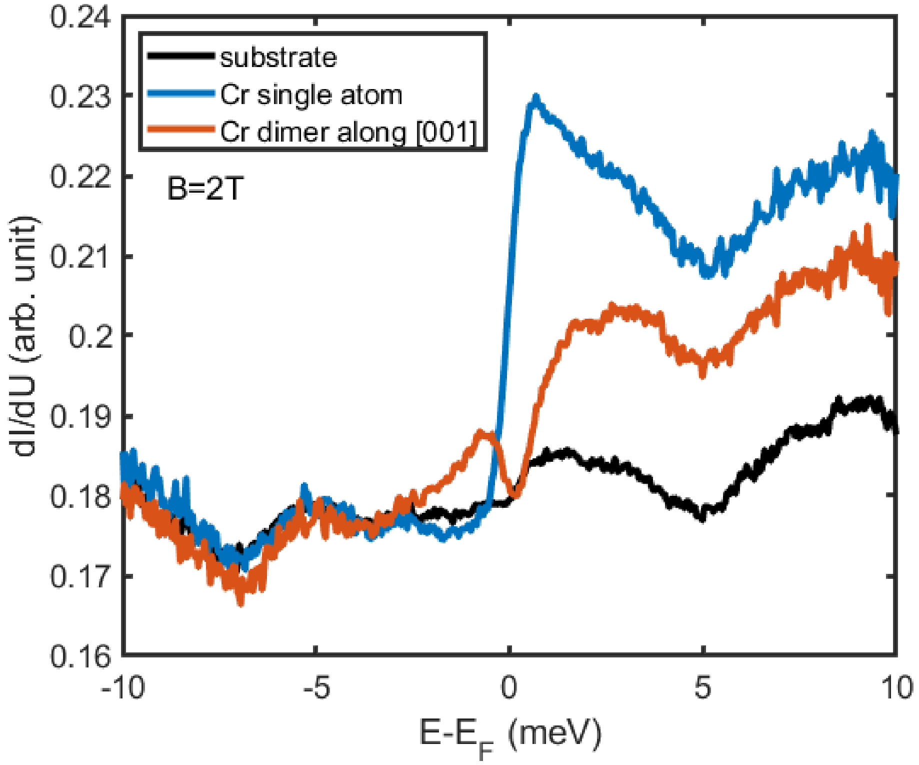

Figure S8: Comparison of raw dI/dU signal acquired on the substrate (black line), a single Cr adatom (blue line), and a dimer aligned along the crystallographic direction [001](red line). All measurements have been acquired using the very same W microtip under a magnetic field of $B = 2$ T applied perpendicular to the sample surface. A dip emerges close to Fermi only for the dimer, an observation which allows to link its origin to the coupling between the adatoms.

Supplementary Note 11: Spin-resolved data acquired using functionalized superconducting tips

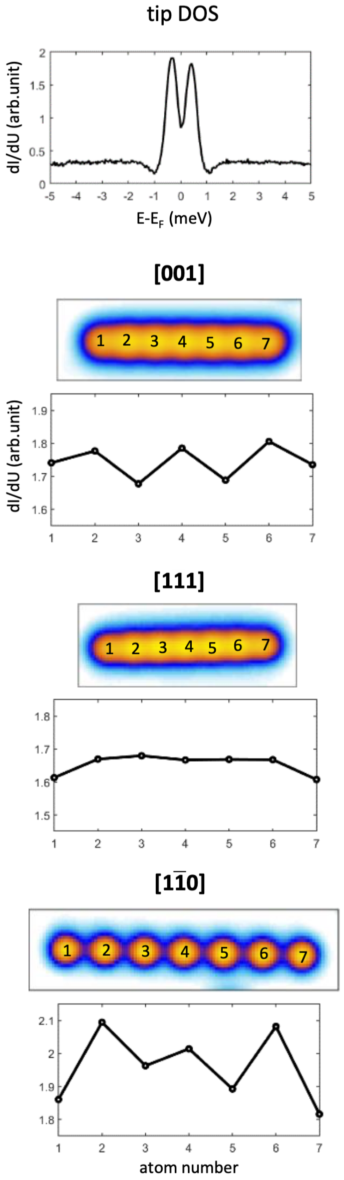

Figure S9: Spin contrast along distinct crystallographic directions visualized using functionalized superconducting tips. An alternating constrast between subsequent adatoms indicative of their AFM coupling is visible along [001] and $[1\bar{1}0]$. A constant amplitude indicative of FM coupling is visible along the $[1\bar{1}1]$ direction. The adatoms at the end of the chains show a distinct signal because of their different local environment. All experimental observations are in line with the magnetic ground state predicted by ab-initio calculations.

Supplementary Note 12: Spectroscopy on progressively longer chains

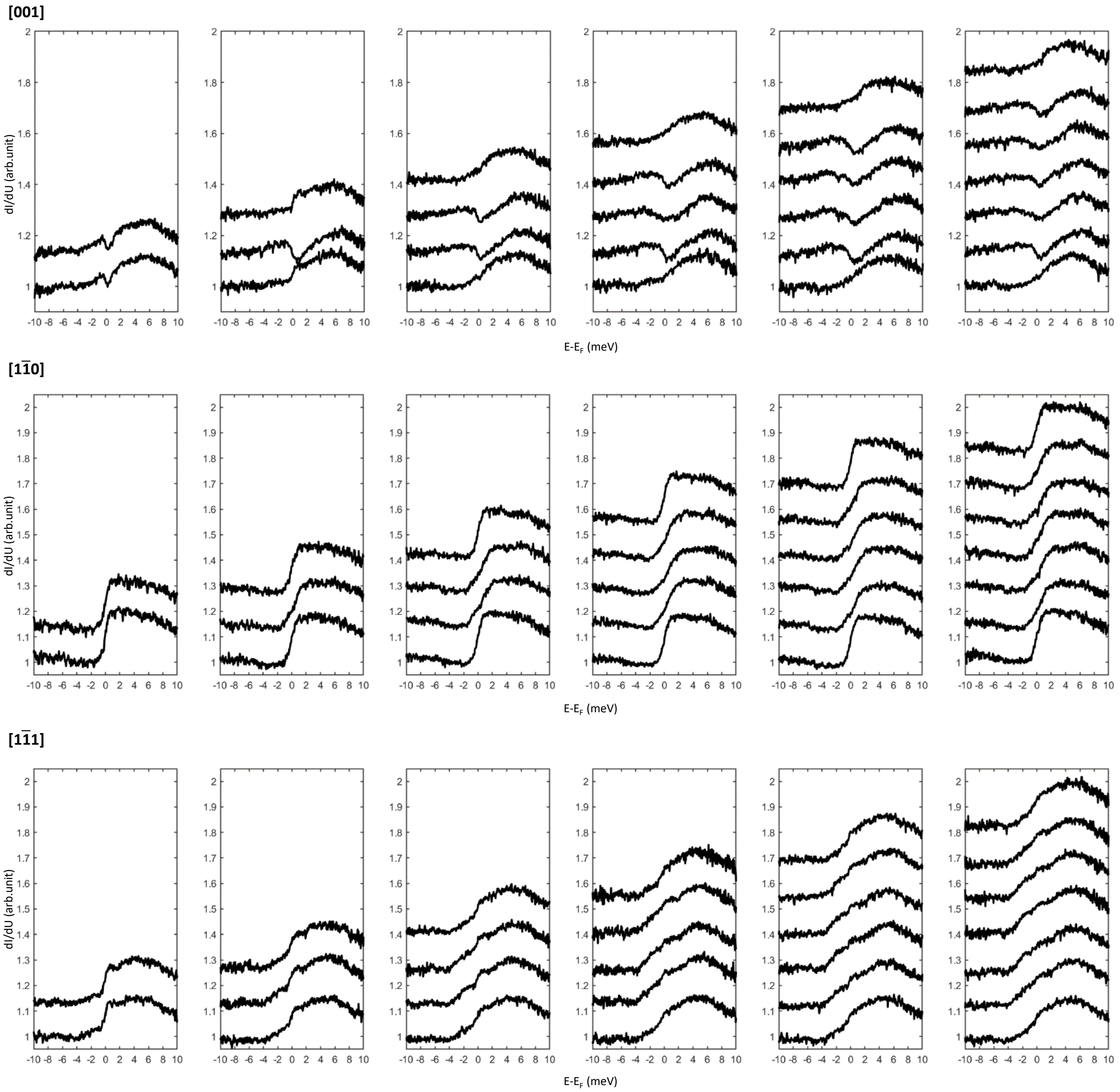

Figure S10: Scanning tunneling spectroscopy on progressively longer chains up to 7 adatoms along the three distinct crystallographic directions scrutinized in the present study. All measurements have been normalized to the substrate and acquired using the very same W microtip under a magnetic field of $B = 2$ T applied perpendicular to the sample surface.

REFERENCES AND NOTES

1. O. Mazurin, Relaxation phenomena in glass. *J. Non Cryst. Solids* **25**, 129–169 (1977).

2. L. C. E. Struik, *Physical Aging in Amorphous Polymers and Other Materials* (Elsevier, 1978).

3. A. J. Kovacs, J. J. Aklonis, J. M. Hutchinson, A. R. Ramos, Isobaric volume and enthalpy recovery of glasses. II. A transparent multiparameter theory. *J. Polym. Sci. B Polym. Phys.* **17**, 1097–1162 (1979).

4. G. W. Scherer, *Relaxation in Glass and Composites* (Wiley, 1986).

5. I. M. Hodge, Physical aging in polymer glasses. *Science* **267**, 1945–1947 (1995).

6. O. S. Narayanaswamy, A model of structural relaxation in glass. *J. Am. Ceram. Soc.* **54**, 491–498 (1971).

7. J. C. Mauro, A. Tandia, K. D. Vargheese, Y. Z. Mauro, M. M. Smedskjaer, Accelerating the design of functional glasses through modeling. *Chem. Mater.* **28**, 4267–4277 (2016).

8. M. Micoulaut, Relaxation and physical aging in network glasses: A review. *Rep. Prog. Phys.* **79**, 066504 (2016).

9. K. Chen, K. S. Schweizer, Molecular theory of physical aging in polymer glasses. *Phys. Rev. Lett.* **98**, 167802 (2007).

10. L. Grassia, S. L. Simon, Modeling volume relaxation of amorphous polymers: Modification of the equation for the relaxation time in the KAHR model. *Polymer* **53**, 3613–3620 (2012).

11. D. Cangialosi, V. M. Boucher, A. Alegria, J. Colmenero, Physical aging in polymers and polymer nanocomposites: Recent results and open questions. *Soft Matter* **9**, 8619–8630 (2013).

12. C. B. Roth, Ed., *Polymer Glasses* (CRC Press, 2017).

13. G. B. McKenna, S. L. Simon, 50th anniversary perspective: Challenges in the dynamics and kinetics of glass-forming polymers. *Macromolecules* **50**, 6333–6361 (2017).

14. S. V. Ketov, Y. H. Sun, S. Nachum, Z. Lu, A. Checchi, A. R. Beraldin, H. Y. Bai, W. H. Wang, D. V. Louzguine-Luzgin, M. A. Carpenter, A. L. Greer, Rejuvenation of metallic glasses by non-affine thermal strain. *Nature* **524**, 200–203 (2015).

15. B. Ruta, E. Pineda, Z. Evenson, Relaxation processes and physical aging in metallic glasses. *J. Phys. Condens. Mat.* **29**, 503002 (2017).

16. S. Küchemann, C. Liu, E. M. Dufresne, J. Shin, R. Maaß, Shear banding leads to accelerated aging dynamics in a metallic glass. *Phys. Rev. B* **97**, 014204 (2018).

17. M. Lüttich, V. M. Giordano, S. Le Floch, E. Pineda, F. Zontone, Y. Luo, K. Samwer, B. Ruta, Anti-aging in ultrastable metallic glasses. *Phys. Rev. Lett.* **120**, 135504 (2018).

18. J. Ketkaew, R. Yamada, H. Wang, D. Kuldinow, B. S. Schroers, W. Dmowski, T. Egami, J. Schroers, The effect of thermal cycling on the fracture toughness of metallic glasses. *Acta Mater.* **184**, 100–108 (2020).

19. S. Vyazovkin, I. Dranca, Effect of physical aging on nucleation of amorphous indomethacin. *J. Phys. Chem. B* **111**, 7283–7287 (2007).

20. F. Bonacci, X. Chateau, E M. Furst, J. Fusier, J. Goyon, A. Lemaitre, Contact and macroscopic ageing in colloidal suspensions. *Nat. Mater.* **19**, 775–780 (2020).

21. J. C. Mauro, *Materials Kinetics: Transport and Rate Phenomena* (Elsevier, 2021).

22. E. Andersen, R. Mikkelsen, S. Kristiansen, M. Hinge, Accelerated physical ageing of poly(1,4-cyclohexylenedimethylene-co-2,2,4,4-tetramethyl-1,3-cyclobutanediol terephthalate). *RSC Adv.* **9**, 14209–14219 (2019).

23. J. Fonseca, T. Gong, L. Jiao, and H.-L. Jiang, Metal-organic frameworks (MOFs) beyond crystallinity: Amorphous MOFs, MOF liquids and MOF glasses. *J. Mater. Chem. A* **9**, 10562–10611 (2021).

24. S. Zhao, Z. Li, C. Zhu, W. Yang, Z. Zhang, D. E. J. Armstrong, P. S. Grant, R. O. Ritchie, M. A. Meyers, Amorphization in extreme deformation of the CrMnFeCoNi high-entropy alloy. *Sci. Adv.* **7**, eabb3108 (2021).

25. S. M. Fielding, P. Sollich, M. E. Cates, Aging and rheology in soft materials. *J. Rheol.* **44**, 323–369 (2000).

26. R. Mandal, P. Sollich, Multiple types of aging in active glasses. *Phys. Rev. Lett.* **125**, 218001 (2020).

27. G. Janzen, L. Janssen, Aging in thermal active glasses. arXiv:2105.05705 [cond-mat.stat-mech] (12 May 2021).

28. R. D. Priestley, Physical aging of confined glasses. *Soft Matter* **5**, 919–926 (2009).

29. L. F. Cugliandolo, J. Kurchan, On the out-of-equilibrium relaxation of the Sherrington-Kirkpatrick model. *J. Phys. A: Math. Gen.* **27**, 5749–5772 (1994).

30. A. P. Holt, D. Fragiadakis, C. M. Roland, Pressure densified 1,3,5-tri(1-naphthyl)benzene glass. I. Volume recovery and physical aging. *J. Chem. Phys.* **151**, 184502 (2019).

31. F. Arceri, F. P. Landes, L. Berthier, G. Biroli, Glasses and aging: A statistical mechanics perspective. arXiv:2006.09725 [cond-mat.stat-mech] (17 June 2020).

32. M. Lulli, C.-S. Lee, H.-Y. Deng, C.-T. Yip, C.-H. Lam, Spatial heterogeneities in structural temperature cause Kovacs' expansion gap paradox in aging of glasses. *Phys. Rev. Lett.* **124**, 095501 (2020).

33. A. Q. Tool, Relation between inelastic deformability and thermal expansion of glass in its annealing range. *J. Am. Ceram. Soc.* **29**, 240–253 (1946).

34. J. C. Dyre, Narayanaswamy's 1971 aging theory and material time. *J. Chem. Phys.* **143**, 114507 (2015).

35. G. B. McKenna, Looking at the glass transition: Challenges of extreme time scales and other interesting problems. *Rubber Chem. Technol.* **93**, 79–120 (2020).

36. T. Hecksher, N. B. Olsen, K. Niss, J. C. Dyre, Physical aging of molecular glasses studied by a device allowing for rapid thermal equilibration. *J. Chem. Phys.* **133**, 174514 (2010).

37. B. Igarashi, T. Christensen, E. H. Larsen, N. B. Olsen, I. H. Pedersen, T. Rasmussen, J. C. Dyre, A cryostat and temperature control system optimized for measuring relaxations of glass-forming liquids. *Rev. Sci. Instrum.* **79**, 045105 (2008a).

38. K. Niss, D. Gundermann, T. Christensen, J. C. Dyre, Dynamic thermal expansivity of liquids near the glass transition. *Phys. Rev. E* **85**, 041501 (2012).

39. K. Niss, Mapping isobaric aging onto the equilibrium phase diagram. *Phys. Rev. Lett.* **119**, 115703 (2017).

40. K. Niss, J. C. Dyre, T. Hecksher, Long-time structural relaxation of glass-forming liquids: Simple or stretched exponential? *J. Chem. Phys.* **152**, 041103 (2020).

41. P. Lunkenheimer, R. Wehn, U. Schneider, A. Loidl, Glassy aging dynamics. *Phys. Rev. Lett.* **95**, 055702 (2005).

42. R. Richert, Supercooled liquids and glasses by dielectric relaxation spectroscopy. *Adv. Chem. Phys.* **156**, 101–195 (2015).

43. P. Lunkenheimer, S. Kastner, M. Köhler, A. Loidl, Temperature development of glassy α-relaxation dynamics determined by broadband dielectric spectroscopy. *Phys. Rev. E* **81**, 051504 (2010).

44. M. Paluch, Z. Wojnarowska, S. Hensel-Bielowka, Heterogeneous dynamics of prototypical ionic glass CKN monitored by physical aging. *Phys. Rev. Lett.* **110**, 015702 (2013).

45. B. Jakobsen, K. Niss, N. B. Olsen, Dielectric and shear mechanical alpha and beta relaxations in seven glass-forming liquids. *J. Chem. Phys.* **123**, 234511 (2005).

46. A. J. Kovacs, Transition vitreuse dans les polymères amorphes. Etude phénoménologique, in *Fortschritte Der Hochpolymeren-Forschung* (Springer, 1964), vol. 3/3, pp. 394–507.

47. T. Hecksher, N. B. Olsen, J. C. Dyre, Fast contribution to the activation energy of a glass-forming liquid. *Proc. Natl. Acad. Sci. U.S.A.* **116**, 16736–16741 (2019).

48. H. N. Ritland, Limitations of the fictive temperature concept. *J. Am. Ceram. Soc.* **39**, 403–406 (1956).

49. L. Song, W. Xu, J. Huo, F. Li, L.-M. Wang, M. D. Ediger, J.-Q. Wang, Activation entropy as a key factor controlling the memory effect in glasses. *Phys. Rev. Lett.* **125**, 135501 (2020).

50. C. Chamon, M. P. Kennett, H. E. Castillo, L. F. Cugliandolo, Separation of time scales and reparametrization invariance for aging systems. *Phys. Rev. Lett.* **89**, 217201 (2002).

51. R. Svoboda, J. Malek, Description of enthalpy relaxation dynamics in terms of TNM model. *J. Non Cryst. Solids* **378**, 186–195 (2013).

52. L. Grassia, D. D'Amore, Constitutive law describing the phenomenology of subyield mechanically stimulated glasses. *Phys. Rev. E* **74**, 021504 (2006).

53. T. Hecksher, N. B. Olsen, J. C. Dyre, Communication: Direct tests of single-parameter aging. *J. Chem. Phys.* **142**, 241103 (2015).

54. A. Sepúlveda, M. Tylinski, A. Guiseppi-Elie, R. Richert, M. D. Ediger, Role of fragility in the formation of highly stable organic glasses. *Phys. Rev. Lett.* **113**, 045901 (2014).

55. D. Cangialosi, V. M. Boucher, A. Alegría, J. Colmenero, Direct evidence of two equilibration mechanisms in glassy polymers. *Phys. Rev. Lett.* **111**, 095701 (2013).

56. X. Monnier, S. Marina, X. Lopez de Pariza, H. Sardon, J. Martin, D. Cangialosi, Physical aging behavior of a glassy polyether. *Polymers* **13**, 954 (2021).

57. S. M. Rekhson, O. V. Mazurin, Stress and structural relaxations in Na_2O-CaO-SiO_2 glass. *J. Am. Ceram. Soc.* **57**, 327–328 (1974).

58. L. E. Reichl, *A Modern Course in Statistical Physics* (Wiley-VCH, ed. 4, 2016).

59. W. Kob, H. C. Andersen, Testing mode-coupling theory for a supercooled binary Lennard-Jones mixture I: The van Hove correlation function. *Phys. Rev. E* **51**, 4626–4641 (1995).

60. J. K. Nielsen, J. C. Dyre, Fluctuation-dissipation theorem for frequency-dependent specific heat. *Phys. Rev. B* **54**, 15754–15761 (1996).

61. T. Körber, R. Stäglich, C. Gainaru, R. Böhmer, E. A. Rössler, Systematic differences in the relaxation stretching of polar molecular liquids probed by dielectric vs magnetic resonance and photon correlation spectroscopy. *J. Chem. Phys.* **153**, 124510 (2020).

62. F. Pabst, J. P. Gabriel, T. Böhmer, P. Weigl, A. Helbling, P. Richter, T. Zourchang, T. Walther, T. Blochowicz, Generic structural relaxation in supercooled liquids. *J. Phys. Chem. Lett.* **12**, 3685–3690 (2021).

63. B. Guiselin, C. Scalliet, L. Berthier, Microscopic origin of excess wings in relaxation spectra of deeply supercooled liquids. arXiv:2103.01569 [cond-mat.soft] (2 March 2021).

64. S. Albert, Th. Bauer, M. Michl, G. Biroli, J.-P. Bouchaud, A. Loidl, P. Lunkenheimer, R. Tourbot, C. Wiertel-Gasquet, F. Ladieu, Fifth-order susceptibility unveils growth of thermodynamic amorphous order in glass-formers. *Science* **352**, 1308–1311 (2016).

65. P. Kim, A. R. Young-Gonzales, R. Richert, Dynamics of glass-forming liquids. XX. Third harmonic experiments of non-linear dielectric effects versus a phenomenological model. *J. Chem. Phys.* **145**, 064510 (2016).

66. J. P. Gabriel, E. Thoms, R. Richert, High electric fields elucidate the hydrogen-bonded structures in 1-phenyl-1-propanol. *J. Mol. Liq.* **330**, 115626 (2021).

67. S. Nosé, A unified formulation of the constant temperature molecular dynamics methods. *J. Chem. Phys.* **81**, 511–519 (1984).

68. N. P. Bailey, T. S. Ingebrigtsen, J. S. Hansen, A. A. Veldhorst, L. Bøhling, C. A. Lemarchand, A. E. Olsen, A. K. Bacher, L. Costigliola, U. R. Pedersen, H. Larsen, J. C. Dyre, T. B. Schrøder, RUMD:

A general purpose molecular dynamics package optimized to utilize GPU hardware down to a few thousand particles. *SciPost Phys.* **3**, 038 (2017).

69. L. A. Roed, T. Hecksher, J. C. Dyre, K. Niss, Generalized single-parameter aging tests and their application to glycerol. *J. Chem. Phys.* **150**, 044501 (2019).

70. B. Igarashi, T. Christensen, E. H. Larsen, N. B. Olsen, I. H. Pedersen, T. Rasmussen, J. C. Dyre, An impedance-measurement setup optimized for measuring relaxations of glass-forming liquids. *Rev. Sci. Instrum.* **79**, 045106 (2008).
